# Integrating *Candida albicans* metabolism with biofilm heterogeneity by transcriptome mapping

**DOI:** 10.1038/srep35436

**Published:** 2016-10-21

**Authors:** Ranjith Rajendran, Ali May, Leighann Sherry, Ryan Kean, Craig Williams, Brian L. Jones, Karl V. Burgess, Jaap Heringa, Sanne Abeln, Bernd W. Brandt, Carol A. Munro, Gordon Ramage

**Affiliations:** 1 School of Medicine, College of Medical, Veterinary and Life Sciences (MVLS), University of Glasgow, UK; 2 Department of Preventive Dentistry, Academic Centre for Dentistry Amsterdam (ACTA), University of Amsterdam and VU University Amsterdam, The Netherlands; 3 Centre for Integrative Bioinformatics VU (IBIVU), VU University Amsterdam, The Netherlands; 4 Institute of Healthcare Associated Infection, School of Health, Nursing and Midwifery, University of the West of Scotland, UK; 5 Microbiology Department, Glasgow Royal Infirmary, Glasgow, UK; 6 Polyomics Facility, MVLS, University of Glasgow, UK; 7 Aberdeen Fungal Group, MRC Centre for Medical Mycology, University of Aberdeen, UK

## Abstract

*Candida albicans* biofilm formation is an important virulence factor in the pathogenesis of disease, a characteristic which has been shown to be heterogeneous in clinical isolates. Using an unbiased computational approach we investigated the central metabolic pathways driving biofilm heterogeneity. Transcripts from high (HBF) and low (LBF) biofilm forming isolates were analysed by RNA sequencing, with 6312 genes identified to be expressed in these two phenotypes. With a dedicated computational approach we identified and validated a significantly differentially expressed subnetwork of genes associated with these biofilm phenotypes. Our analysis revealed amino acid metabolism, such as arginine, proline, aspartate and glutamate metabolism, were predominantly upregulated in the HBF phenotype. On the contrary, purine, starch and sucrose metabolism was generally upregulated in the LBF phenotype. The aspartate aminotransferase gene *AAT1* was found to be a common member of these amino acid pathways and significantly upregulated in the HBF phenotype. Pharmacological inhibition of AAT1 enzyme activity significantly reduced biofilm formation in a dose-dependent manner. Collectively, these findings provide evidence that biofilm phenotype is associated with differential regulation of metabolic pathways. Understanding and targeting such pathways, such as amino acid metabolism, is potentially useful for developing diagnostics and new antifungals to treat biofilm-based infections.

*Candida* bloodstream infections (CBSI) are the third most frequent infection in intensive care units and are associated with unacceptable rates of morbidity and mortality (up to 50%)^1^,^2^, as well as high healthcare costs^3^,^4^,^5^. The presence of indwelling devices, such as central venous catheters, profoundly impacts these associated problems. Moreover, poor patient prognosis is impacted by delayed diagnostics and limited antifungal options^6^. The recalcitrant nature of biofilms reduces the effectiveness of antifungal regimens, thereby complicating the clinical management of CBSI^7^. *Candida albicans* is the most prevalent and pathogenic of the *Candida* species involved in CBSI, which is typified by its capacity to form robust biofilms. Therefore, identifying the molecular mechanisms that contribute to *C. albicans* biofilm formation and developing new strategies to prevent CBSI is vital for minimising mortality rates and healthcare costs.

*C. albicans* is an opportunistic pathogen that frequently uses the biofilm lifestyle as a mode of protection against the host immune system and chemotherapeutic intervention. *C. albicans* capacity for physiological adaptation permits nutrient assimilation and growth in a variety of host environments^8^. A key component of *C. albicans* adaptation is filamentous growth, which is stimulated by a variety of environmental and physiological factors, including the carbohydrate source, amino acid starvation, hypoxia, elevated levels of CO_2_, pH and temperature^8^,^9^,^10^. Metabolic adaptation to such conditions, as well as antifungal agents and stress conditions, promote *C. albicans* pathogenicity, which includes yeast-hypha morphogenesis, phenotypic switching, adhesins, invasins, and secreted hydrolases^8^, factors all important to the biofilm phenotype^11^. In fact, these biofilm related processes are driven by six key transcriptional regulators as part of a complex transcriptional circuitry^12^. While this level of global control is understood, how these relate to central metabolic processes is limited. Although several anabolic and catabolic pathways, such as those involved in carbon and amino acid metabolisms, have been shown to play a pivotal role in *C. albicans* pathogenicity, the key pathways associated with biofilm formation remain unclear^13^,^14^.

It has recently been shown that biofilm forming ability of candidaemia isolates, and the choice of antifungal drug used for treatment, is significantly associated with patient mortality^15^,^16^. Subsequent studies by our group identified that the biofilm phenotype is heterogeneous, and stratification of clinical isolates as either high- or low- biofilm formers (HBF and LBF) has been shown to be a good predictor of clinical outcomes, where the HBF were shown to significantly correlate with mortality, unlike LBF^17^. While significant and elegant efforts have been made to unravel the mechanisms of biofilm formation^12^, these have been limited to laboratory strains, which have limited resemblance to the biological heterogeneity exhibited by clinical isolates^16^,^17^. Therefore, there is solid rationale to use clinically derived strains for studies that can explain why certain clinical *C. albicans* biofilms grow to be much more pathogenic (HBF) than others (LBF).

In this study, we evaluated the hypothesis that metabolic adaptation plays an important role in *C. albicans* biofilm heterogeneity, where differential expression of certain *C. albicans* genes or pathways is the primary factor that separates the LBF from the HBF. To do so, we performed a comparative RNA sequencing (RNA-Seq) analysis on samples derived from the bloodstream isolates of candidaemia patients, which were previously identified to be HBF or LBF. To put our results from the differential expression analysis into a biologically-interpretable and metabolic context, we first created a global network of interacting *C. albicans* genes by combining all *C. albicans* pathway maps in the KEGG database^18^. In this global network, in order to highlight the region that shows the strongest differential expression, we used a bioinformatics workflow that identifies the subnetwork of the most significantly differentially expressed genes between the LBF and HBF. This network-based approach has been shown to recover relevant biological pathways that were significantly deregulated in single-species as well as multi-microbial communities^19^,^20^.

To validate our computational findings experimentally, we selected four genes from the identified subnetwork, and performed real-time quantitative PCR (qPCR) experiments. Using qPCR, the increase or the decrease in the expression of these four genes in the HBF was confirmed using a different set of clinical isolates. Furthermore, we performed an inhibition experiment using the *AAT1* gene, a central node in the subnetwork involved in several amino acid pathways, to evaluate the effect of its inhibition on biofilm formation and *C. albicans* survival. Overall, this study addresses a major gap in our understanding of the metabolic pathways and their role in clinically important *C. albicans* biofilm entity. Elucidating this essential element of pathogenesis provides invaluable information to identify metabolic biomarkers for early biofilm diagnostics and paves the way for the development of personalised therapeutic strategies.

## Results

### RNA-Seq analysis

**Figure 1:**
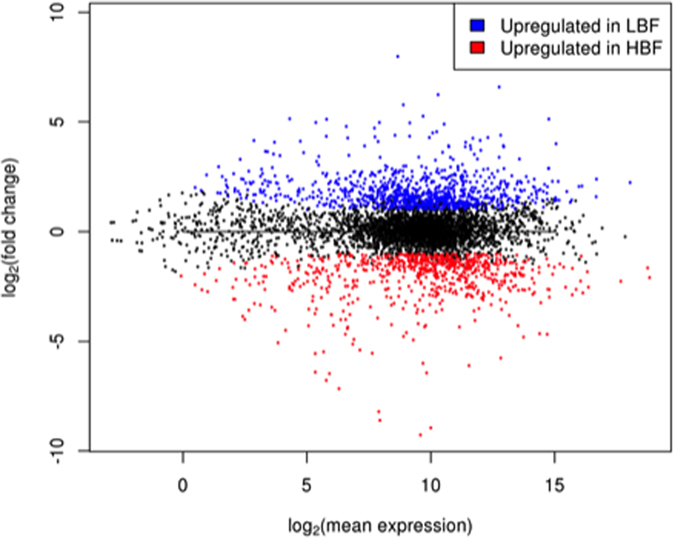
Differential expression of *C. albicans* genes in the low (LBF) and high biofilm formers (HBF) groups. Log fold changes are plotted against the log mean expression of 6312 *C. albicans* genes. The blue and red dots denote the genes that were upregulated by a minimum 2-fold change in expression (*P* < 0.05) in the LBF (1007) and HBF (783) respectively.

In order to identify the differentially expressed genes between the *C. albicans* LBF and HBF, we performed a comparative RNA-Seq analysis on pre-characterised clinical isolates^17^. Sequencing the transcriptome of these six samples (each isolate in triplicate) resulted in nearly 141 million raw RNA-Seq reads (Table 1). Following quality control and filtering reads from non-coding RNAs, a high percentage of raw reads was mapped to the *C. albicans* genome (87.4% and 95% of LBF and HBF reads, respectively). The percentage of reads that were mapped to the regions of the genome annotated with KEGG genes^21^, was also high in both LBF (66%) and HBF (74%), indicating that the coding genome regions were mostly covered with the genes in our database. Out of 7281 genes, 6312 were found to be expressed in both conditions, among which 1007 and 783 were significantly upregulated (*P* < 0.05) with a minimum 2-fold change in the LBF and HBF, respectively (Fig. 1).

### An overview of differentially expressed functions

To obtain an overview of functional differences, we categorised the genes that showed a minimum 2-fold upregulation (*P* < 0.05) in their expression in the LBF (1007) and HBF (783) into Gene Ontology processes^22^ (Supp. Fig. 1). These functional categories included a range of processes including filamentous growth, biofilm formation, cellular transport, response to stress, and pathogenesis.

In both the LBF and HBF, the biofilm formation process represented only 4% of the number of significantly upregulated genes (Supp. Fig. 1). However, when certain genes in this functional category were investigated, those associated with a yeast state were found to be upregulated in the LBF (*YWP1*, log_2_ fold change = 8.6; *NRG1*, log_2_ fc = 3.7), whereas the HBF had hyphal specific gene expression increased (*HWP1*, log_2_ fc = 6; *HYR1*, log_2_ fc = 3.8; and *ALS3*, log_2_ fc = 2.8). These expression patterns are consistent with the yeast and hyphal phenotypes presented in LBF and HBF, respectively. Similar to the biofilm formation process, there was no difference in the percentage of upregulated genes associated with cell adhesion and hyphal growth in the LBF and HBF, which in total accounted for only 4% of upregulated genes in each group.

The three functional groups that contained the greatest number of significantly upregulated genes were transport, response to chemicals, and response to stress, which accounted for 52% (LBF) and 48% (HBF) of the total number of genes analysed for this section (1790). In the LBF, the greatest change in expression, namely 21%, was found in genes related to stress response (Fig. 1B). Genes associated with cellular transport were the mostly up-regulated ones in the HBF, accounting for 20% of genes (Supp. Fig. 1). A common limitation of differential gene expression analyses is the human bias for investigating the genes related to preconceived hypotheses and those reported in the existing literature. An expected finding was the higher expression of *HWP1* in the HBF, as this is a hyphal-specific gene known to be involved in biofilm formation^23^,^24^. Reciprocally, the yeast wall protein (*YWP1*) was significantly upregulated in the LBF isolates. Cells lacking this are more adhesive and can form thicker biofilms^25^. Moreover, we identified *ECE1* was significantly upregulated (log_2_ 6-fold) in the HBF, which codes for the novel candidalysin peptide toxin that has recently been reported to induce epithelial cell damage^26^. *SAP8* was also upregulated in the HBF phentotype, which is a protease previously identified to be highly expressed in mature biofilms of denture stomatitis patients^27^. Additionally, a glucose transporter gene, *HGT9,* was also upregulated in the HBF, and from studies we know that glucose has a direct effect on *C. albicans* adhesion and biofilm formation^28^. Therefore, the upregulation of *HGT9* expression found in this study may be due to the increased utilisation of carbon sources in HFB isolates. Despite these potential genes of interest, we identified a large number of other genes (n = 1790) that were differentially expressed between the LBF and HBF, which is in line with other RNA-Seq studies looking at differentially expressed genes in *C. albicans* planktonic cells compared to those grown in a biofilm (n = 1599)^12^. While we can manually try and make sense of the biological data one gene at a time, our ability to decipher what the global impact of all the changes mean in the context of one another is limited.

In this analysis, many genes are grouped into a single functional category and some genes are even associated with multiple categories, making this a very coarse-grained approach. The *HWP1* example shows that only looking at the high level categories does not help gain an understanding of the key players involved, and may indeed lead to a biased approach to analysis through selection of perceived biofilm related genes. Hence, this type of analysis is less suitable for pinpointing the most important factors in the metabolic network that contribute to biofilm heterogeneity.

### Network analysis identifies the significant changes

In order to obtain a more informative, accurate picture of the mechanisms involved in *C. albicans* biofilm formation, we performed a computational analysis that takes into account the full network of *C. albicans* pathways present in the KEGG database. This approach is able to identify the most significantly differentially expressed parts in this full network, and can be summarised in three main steps: i) constructing a global pathway map of interacting genes, ii) assigning a positive or a negative numerical score to each gene in the network, which reflects the significance of the gene’s differential expression, and iii) applying a search algorithm on the score-annotated network for identifying the maximum-scoring subnetwork. This subnetwork corresponds to the region in the global network where the (interacting) genes with the most significant differential expression are located.

To this end, we integrated 104 *C. albicans*-specific KEGG pathway maps that contain the molecular interaction and reaction gene networks for functional categories, including metabolism, genetic and environmental information processing and cellular processes into a single, global *C. albicans* gene network (2960 genes). After removing the genes that did not interact with any other genes from the network, the global network consisted of 1721 nodes (genes) and 8197 undirected edges (gene interactions).

**Figure 2:**
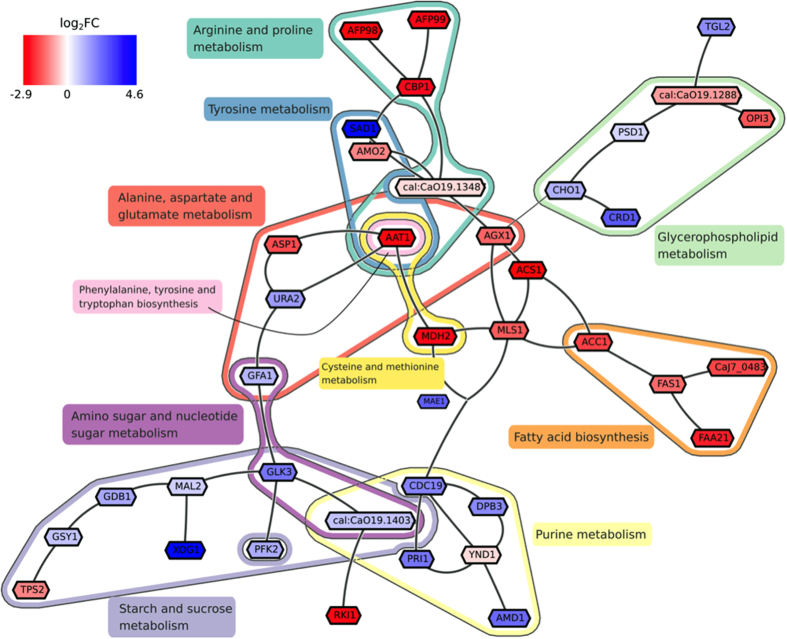
The maximum-scoring subnetwork identified in the global *C. albicans* metabolic network. The subnetwork corresponds to the region in the global metabolic gene network where the most significantly differentially expressed genes between the LBF and HBF are located. The colours red and blue with gene names denote the fold change (upregulation in HBF and LBF, respectively). Different coloured lines indicate different metabolic pathways (labelled near each pathway). This computationally-identified subnetwork suggests the aspartate aminotransferase (*AAT1*) to be a key player in biofilm heterogeneity. Moreover it highlights the importance of amino acid metabolic pathways.

Taking the overlap between the genes that were expressed in both LBF and HBF samples with those in the global *C. albicans* network resulted in a final network of 859 genes and 1997 gene interactions. Next, we used a strict *P* value threshold of 10^−10^: genes in the network with smaller *P* values were assigned positive scores, while genes with larger *P* values were assigned negative scores (see Methods). Correspondingly, we assigned 59 of 859 genes in the network positive (node) scores, while the rest of the genes attained negative scores. The Heinz network search algorithm was applied on the network to calculate the maximum-scoring sub-network as described elsewhere^20^. The identified subnetwork consisted of 39 genes (Supp. Table), of which 20 were down- and 19 were upregulated in the HBF (Fig. 2). The total number of pathways associated with these genes was 41 (Table 2), among which (i) arginine and proline metabolism, (ii) pyruvate metabolism and (iii) fatty acid metabolism were predominantly upregulated in the HBF samples (Fig. 2). In contrast, purine metabolism and starch and sucrose metabolism were mostly up-regulated in the LBF samples (Fig. 2).

### Validation of RNA-Seq results

**Figure 3:**
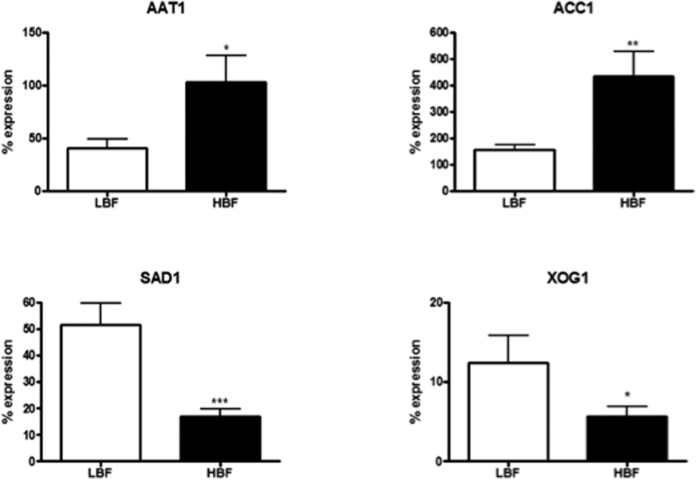
Validation of RNA-seq data. Expression of selected genes (*AAT1, ACC1, SAD1* and *XOG1*) was assessed by qPCR analysis of RNA isolated from 24 h biofilms of 9 HBF and 9 LBF clinical isolates. Graphs show percentage expression of each gene compared to a housekeeping gene *ACT1*. *P < 0.05, **P < 0.01, ***P < 0.001.

To validate the findings from our RNA-Seq analysis, we performed real-time quantitative PCR (qPCR) on 9 additional HBF and LBF isolates for four genes, *AAT1, ACC1, SAD1* and *XOG1*, which are members of the key KEGG pathways that were found by our network analysis (Fig. 3). Two of the selected genes, *SAD1* and *XOG1*, involved in amino acid and sugar metabolism, respectively, were significantly upregulated (*P* < 0.05) in the LBF. *AAT1* and *ACC1*, involved in amino acid and fatty acid metabolism, respectively, were upregulated (*P* < 0.05) in the HBF. For all selected genes, qPCR results were in agreement with the results from the RNA-seq analysis (Fig. 3): the expression levels of *SAD1* and XOG1 were found to be 3-fold (*P* < 0.001) and 2-fold (*P* < 0.05) upregulated in the LBF. Furthermore, both *AAT1* and *ACC1* were found to be significantly upregulated by 3-fold in the HBF (*P* < 0.05 and *P* < 0.01, respectively).

### AAT1 drives the biofilm phenotype

**Figure 4:**
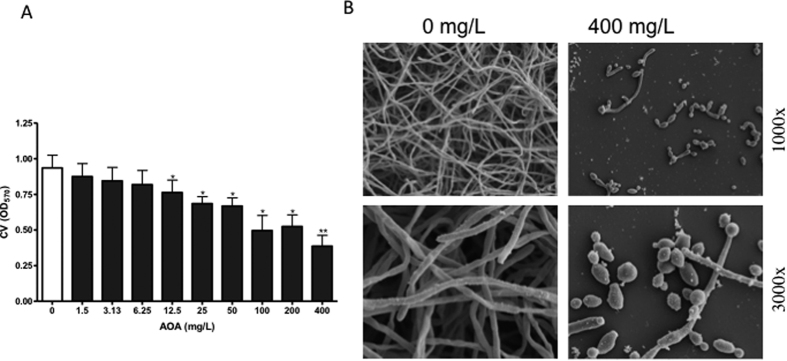
Inhibition of AAT activity reduces biofilm formation. *C. albicans* biofilms (n = 6) were formed in the presence of serially diluted AOA (Aminoxy acetate [AAT inhibitor]), concentration range from 0–400 mg/L. After 24 h incubation, biofilm biomass was assessed by crystal violet (CV) assay. (**A**) Graph shows CV absorbance. *P < 0.05, **P < 0.01. (**B**) Biofilms grown on thermonox coverslips in the presence or absence of 400 mg/L of AOA were fixed and processed for scanning electron microscope imaging. Micrographs show the biofilm phenotype at 1000x and 3000x magnification.

**Figure 5:**
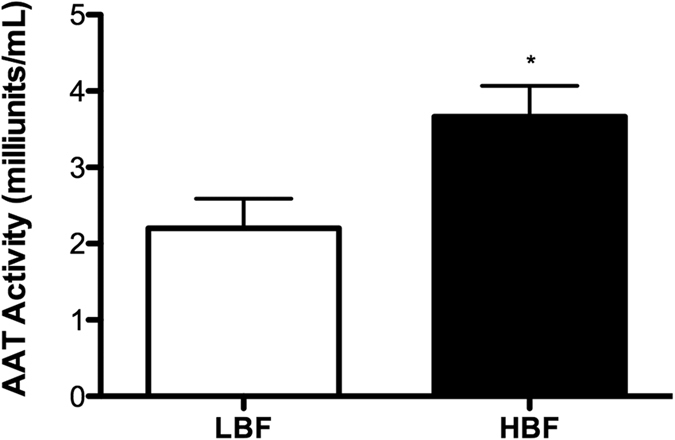
Biochemical validation of AAT enzyme activity levels in HBF and LBF isolates. Levels of the AAT enzyme activity in 24 h grown HBF (n = 5) and LBF (n = 5) biofilms were assessed using a colorimetric assay. The bar graph shows AAT activity standardised to biofilm biomass. *P < 0.05.

The maximum-scoring subnetwork identified by our bioinformatic workflow highlighted an overall upregulation of genes in different amino acid metabolism pathways in the HBF, therefore, these genes were investigated further. The gene *AAT1*, coding for aspartate aminotransferase, which plays a central role in different amino acid metabolism pathways, was found to be significantly upregulated in the HBF (Fig. 2). The impact of inhibition of the AAT enzyme activity by the aminoxy acetate (AOA) inhibitor on *C. albicans* biofilm formation was then examined. At all tested concentrations, AOA had no toxic effect on *C. albicans* growth. Next, biofilm formation by the HBF (n = 6) in the presence of different concentrations of AOA was assessed. There was significant reduction in biofilm formation in the presence of AOA in a dose dependant manner (Fig. 4A), confirming an apparent role of AAT in biofilm formation. Conversely, no significant reduction in biofilm biomass was found with LBF isolates (n = 5) in the presence of AOA (Supp. Fig. 3). Analysis of the biofilm phenotype by scanning electron microscopy showed striking differences with more yeast and pseudohyphal cells with AOA treatment compared to the filamentous multidimensional architecture in untreated controls (Fig. 4B). This suggests that AAT plays a role in modulating biofilm formation via regulating filamentation and morphogenesis in *C. albicans*. Furthermore, biochemical assessment of secreted AAT levels with different clinical isolates confirmed that a significantly higher level of AAT was produced by HBF compared to LBF biofilms (Fig. 5).

## Discussion

Understanding the molecular mechanisms involved in the phenotypic differences between *C. albicans* clinical isolates is an important avenue of investigation for developing and improving clinical management strategies, both in terms of antifungal therapy and the development of new diagnostic strategies. We previously reported that *C. albicans* biofilm heterogeneity is an important clinical entity^17^, and that extracellular DNA (eDNA) release contributes to biofilm-associated antifungal resistance^29^. However, this somewhat targeted investigational approach fails to take into account the global factors driving these paradoxical phenotypes that we observe clinically. Here, we identified the differential expression of several metabolic pathways that potentially contribute to *C. albicans* biofilm heterogeneity. This study is the first to use an integrated KEGG pathway analysis to reveal biologically important pathways in *C. albicans,* which has highlighted the importance of aspartate amino transferase in *C. albicans* biofilm development, and identified its potential as a therapeutic target for improving clinical management.

In order to avoid this limitation and focus on novel, promising, targets for improving the management of biofilm-based infections, we conducted an advanced network-based analysis. Using this approach, we found various amino acids, pyruvate and fatty acid metabolism pathways to be central pathways upregulated in the HBF. The increased intracellular level of pyruvate, pentoses and amino acids in early biofilm phase contributes to increased biomass in maturation phase^30^. Fatty acids have diverse functions in the cells as they are important constituents of lipids, plays a role in energy storage, integrity and dynamics of cell membrane and adhesion to host cells^31^. Conversely, purine, starch and sucrose metabolic pathways were predominantly expressed in LBF.

Various amino acid pathways, including arginine, proline, purine, alanine, aspartate and glutamate metabolisms were shown to be considerably upregulated in the HBF. The exact regulatory mechanism behind this amino acid metabolism is not yet clear. It is known that amino acid starvation activates the general amino acid control (GCN) response, and that *GCN4*-regulated amino acid metabolism is required for normal biofilm formation by *C. albicans*^13^. In response to amino acid starvation, *GCN4* regulates a number of genes, which in turn activates amino acid biosynthesis and morphogenesis in *C. albicans* and *Saccharomyces cerevisiae*^9^. Interestingly, the *AAT1* gene that encodes aspartate aminotransferase, was found to be a key member of different amino acid pathways that were upregulated in the HBF. AAT1 was shown to be a soluble protein in hyphae, and knocking out *AAT1* gene does not impact *C. albicans* viability^32^,^33^. The mechanism of activation of *AAT1* and its role in biofilm formation is not yet clear to us, but we hypothesise that this acts downstream of the *GCN4* pathway. Our data suggests that it may have residual morphological effects, as also reported for *GCN4*^9^, and this may interfere with biofilm stability as a result. However, further mechanistic studies are required to confirm this. Nevertheless, pharmacological inhibition of AAT was shown to significantly reduce the *C. albicans* biofilm biomass, confirming its apparent role in biofilm formation and its potential as an antifungal target. Our biochemical assay showed a significantly higher AAT level (*P* < 0.05) among HBF isolates compared to LBF, suggesting that AAT has the potential to be used diagnostically to differentiate the two phenotypes.

Collectively, using an integrated approach to analyse clinically important *C. albicans* isolates, we were able to link various metabolic pathways to defined biofilm phenotypes. A dedicated computational approach allowed us to dissect high-dimensional RNA-Seq output and to recover the essential pathway biology. The genes that were identified to be the key components were subsequently confirmed (*AAT1, ACC1, SAD1* and *XOG1*) and validated (*AAT1) in vitro* experiments. *AAT1* and *ACC1* were found to be upregulated in HBF and *SAD1* and *XOG1* were upregulated in LBF. Previous studies have investigated the role of some of these genes in biofilm formation. For example, *XOG1* gene shown to have no impact on cell wall glucan content of biofilm cells, nor it is necessary for filamentation or biofilm formation^34^, which is in agreement with our data. *ACC1* encodes acetyl-coenzyme-A carboxylases, which have been shown to be upregulated in biofilm cells. Its transcription may indirectly depend on oxygen availability, but also on the availability of inositol in *S. cerevisiae*^35^.

Our ultimate aim from these studies and moving forward is to identify a potential target for biofilm diagnostics and antifungal development. Here, we highlighted the importance of amino acid metabolism in the HBF. The key enzyme of this metabolism, *AAT*, was also shown to be a potential target for the management of biofilm-based infections. Future integrated OMICs approaches will support these investigations.

## Materials and Methods

### Culture conditions and standardisation

This study utilised *Candida albicans* strain SC5314 and a series of routine patient anonymised clinical bloodstream isolates (n  =  30) collected under the approval of the NHS Scotland Caldicott Gaurdians, as part of candidaemia epidemiology surveillance study^17^,^36^. *C. albicans* clinical bloodstream isolates previously identified to have high biofilm formation (HBF [n = 15]) and low biofilm formation (LBF [n = 15]) were used throughout this study^17^,^36^. Isolates were stored in Microbank^®^ vials (Pro-Lab Diagnostics, Cheshire, UK) at −80 °C until sub-cultured onto Sabouraud’s dextrose agar (SAB [Sigma-Aldrich, Dorset, UK]). Isolates were propagated in yeast peptone dextrose (YPD) medium (Sigma-Aldrich, Dorset, UK), washed by centrifugation and re-suspended in RPMI-1640 (Sigma-Aldrich, Dorset, UK) to 1 × 10^6^ cells/mL, as described previously^37^. Biofilms were grown in polystyrene plates or 75 cm^2^ tissue culture flasks in RPMI for 24 h at 37 °C.

### RNA extraction and sequencing

Following incubation, biofilms were washed with PBS before a cell scraper was used to dislodge the biomass, which was homogenised using a bead beater, and RNA extracted using the TRIzol™ (Life Technologies, Paisley, UK) method as described previously^38^. Total RNA was DNase (Qiagen, Crawley, UK) treated and purified using an RNeasy MiniElute clean up kit (Qiagen, Crawley, UK), as per manufacturer’s instructions. RNA was quantified and quality assessed using a NanoDrop spectrophotometer (ND-1000, ThermoScientific, Loughborough, UK). The integrity of the RNA was assessed using an agarose gel to visualise the two distinct rRNA bands. Each isolate was grown in triplicate and a minimum of 10 μg of total RNA was submitted for each sample and sent for sequencing to The GenePool (Edinburgh, UK). RNA integrity was assessed using a Bioanalyzer where an RIN value > 7.0 was deemed acceptable for RNA-Seq using Illumina 50 base pair sequencing.

### Pre-processing of RNA-Seq data

The reads that had an overlap of at least 8 bp with the adapter sequence (Illumina TruSeq v3 i7) were filtered out using cutadapt v1.7.1 with default parameters^39^. Quality-trimming on the remaining reads was done using sickle v1.31 (available at https://github.com/najoshi/sickle) with an average quality score of 15 over a 5-bp sliding window. Reads that were trimmed by sickle to a length shorter than 30 bp were discarded. Subsequently, reads originating from non-coding RNAs were filtered out using sortMeRNA v1.99-beta^40^ with default parameters and databases.

### Candida albicans genome and its annotation

The diploid genome sequence of *Candida albicans* SC5314 (assembly 22) was downloaded (February 2015) from the *Candida* Genome Database^45^, http://www.candidagenome.org/and was converted to a haploid genome by discarding one of the chromosomes in each homologous chromosome pair. This haploid reference was annotated using the *C. albicans* genes in the KEGG database^21^ as follows: (i) The nucleotide sequences of 14,661 *C. albicans* SC5314 genes in the KEGG database were clustered at 85% sequence identity using the USEARCH^41^ algorithm (-cluster_fast option). The resulting 7,281 non-homologous genes were mapped to the haploid *C. albicans* genome using the UBLAST algorithm (*E*-value 10^−6^, query coverage 0.95) to annotate the RNA-coding regions in the genome in the form of a GTF file using in-house scripts. Subsequently, STAR RNA-Seq aligner^42^ was used to generate index files for the haploid genome using the GTF annotations. The pre-processed reads were then aligned to the indexed genome using the STAR RNA-Seq aligner with default parameters.

### Differential expression analysis

Following mapping, for each sample, the number of reads that were unambiguously mapped to a gene was estimated by parsing the STAR RNA-Seq alignment output by HTSeq^43^ v.0.6.1 (default parameters). Next, an unpaired differential expression analysis (LBF vs HBF) using DESeq2 v1.6.1^44^ was performed with default settings as described in the package vignette. Briefly, the package normalizes read counts to account for different library sizes and uses negative binomial generalized linear models to determine a conservative set of genes that are differentially expressed across experimental conditions.

### Functional annotation

We determined the Gene Ontology^22^ functional processes of significantly upregulated genes that showed a minimum 2-fold increase in their expression in the HBF and LBF. To do so, the list of genes were used as input for the GO Slim Mapper tool of the *Candida* Genome Database^45^ with the preselection of the following processes: biofilm formation, cell adhesion, cell budding, cell wall organization, cellular homeostasis, filamentous growth, hyphal growth, interspecies interaction between organisms, pathogenesis, pseudohyphal growth, response to chemical, response to drug, response to stress, signal transduction and transport.

### C. albicans gene network

The KEGG pathway maps of *C. albicans* SC5314 were used to create a global functional interaction network of *C. albicans* genes. To this end, gene relations defined in the individual pathways, such as acting on the same substrate, were used to derive the interactions between genes, which were then merged into a single network. The global gene network for the HBF-LBF comparison was then obtained by taking the intersection of genes in the global *C. albicans* network and those genes that were tested for differential expression, followed by the removal of non-interacting genes.

### Identifying the key metabolic subnetwork

Following the works of Dittrich, *et al*.^46^ and May, *et al*.^47^, the computational analysis presented here aims at identifying the set of connected (i.e. interacting) *C. albicans* genes with the most significant deregulation across the LBF and HBF groups. In other words, the result is a subnetwork within the global *C. albicans* network, which contains the connected genes of most significant fold changes. This requires (i) the annotation of genes (nodes) in the global network with positive and negative numerical scores that reflect the significance of the difference in expression across conditions, followed by (ii) the application of a network search algorithm that can identify the maximum-scoring region in the network. In order to perform these steps, first, we used the BioNet R package^48^ to fit a statistical model to the *P* value distribution of fold changes (see Supp. Fig. 2., for details, see Pounds and Morris^49^). Next, a false-discovery rate of 10^−9^ was used to determine a *P* value threshold (10^−10^), according to which, the statistical model was used to calculate positive and negative weights for genes with *P* values smaller and larger than the threshold, respectively. Once the genes (nodes) in the network were annotated with such numerical scores, the Heinz algorithm^46^ was used to calculate the maximum-scoring subnetwork that delineated the most significantly deregulated region in the larger network. The subnetwork was visualised using the Cytoscape^50^ plugin eXamine^51^.

### Validation of the RNA-Seq analysis

*C. albicans* clinical isolates exhibiting LBF (n  =  9) and HBF (n  =  9) were selected for the analysis of genes related to biofilm formation. Biofilms were grown and RNA was extracted as described elsewhere^36^. Next, cDNA was synthesised using High Capacity RNA to cDNA kit (Life Technologies, Paisley, UK) in a MyCycler PCR machine (Bio-Rad Laboratories, Hertfordshire, UK), following manufacturer’s instructions. The primers for the four selected genes from the KEGG pathway analysis are listed in Table 3. Cycle conditions consisted of 2 min at 50 °C, 10 min at 95 °C and forty cycles of 15 s at 95 °C and 60 s at 60 °C. All qPCR assays were performed in duplicate using MxProP Quantitative PCR machine and MxProP 3000 software (Stratagene, Amsterdam, Netherlands) and controls consisted of reactions in which reverse transcriptase template were absent. Gene expression was calculated using the ΔCt method where the genes of interest were normalised to the housekeeping gene *ACT1*.

### Validation of aminotransferase activity

Aspartate amino transferase enzyme activity was measured using a commercial assay kit (Sigma, Dorset, UK), by following the manufacturer’s instructions. To assess the impact of AAT inhibition on *C. albicans* biofilm formation, we used an inhibitor called aminoxyacetate (AOA [Sigma, Dorset, UK]). *C. albicans* clinical isolates (n = 12) were grown for 24 h in the presence of serially diluted concentration of AOA (ranging from 0 to 400 mg/L) in RPMI at 37 °C, with an untreated control included on each plate for comparison. After incubation, the biofilms were washed in PBS and their biomasses were quantified using a crystal violet technique previously described by our group^17^. Growth curve analysis was also performed for isolates grown in the presence of AOA (100 and 400 mg/L) by measuring absorbance at a wavelength of 530 nm for 24 h in YPD and RPMI media. No significant change in endpoint growth levels were found in the presence of AOA at any tested concentrations, though a slight delay in growth rate in RPMI (data not shown).

### Scanning Electron microscopy

*C. albicans* type strain SC5314 was grown directly onto Thermanox™ coverslips (Nunc, Roskilde, Denmark) for 24 h in the presence and absence of AOA (400 mg/L) After incubation period, biofilms were carefully washed with PBS and processed for SEM as previously described^52^. Briefly, samples were fixed in 2% paraformaldehyde, 2% glutaraldehyde, and 0.15% [wt/vol] alcian blue in 0.15 M sodium cacodylate (pH 7.4). The biofilms were sputter coated with gold and viewed under a JEOL JSM-6400 scanning electron microscope.

## Additional Information

**How to cite this article**: Rajendran, R. *et al*. Integrating *Candida albicans* metabolism with biofilm heterogeneity by transcriptome mapping. *Sci. Rep.* **6**, 35436; doi: 10.1038/srep35436 (2016).

## Supplementary Material

Supplementary Information

## Figures and Tables

**Table 1:** The vast majority of reads in both low (LBF) and high biofilm formers (HBF) passed the quality control.

	Nr. of samples	Nr. of raw reads	% of reads after QC	% reads after t/rRNA removal	% mapped reads to genome	% mapped reads to KEGG genes
All samples	6	140,631,025	99.32	97.80	91.40	70.34
LBF	3	68,484,992	98.75	96.14	87.40	66.21
HBF	3	72,146,033	99.86	99.38	95.20	74.26

A large portion was mapped to the KEGG genes that were used to annotate the *C. albicans* genome. Percentages are relative to the number of raw reads.

**Table 2:** KEGG pathways that are associated with the genes in the maximum-scoring sub network.

KEGG Pathway ID	Cumulative pathway score	Pathway name	Gene IDs
cal00330	198.9823	Arginine and proline metabolism	cal:CaO19.13487,AAT1,CBP1,AFP99,AFP98
cal00230	132.1469	Purine metabolism	YND1, AMD1, CDC19, PRI1, DPB3, cal:CaO19.14031,
cal00500	110.8155	Starch and sucrose metabolism	GLK3, MAL2, GSY1, TPS2, GDB1, cal:CaO19.14031, XOG1
cal00410	104.9783	beta-Alanine metabolism	cal:CaO19.13487, CBP1, AMO2
cal00250	59.83122	Alanine, aspartate and glutamate metabolism	ASP1, AAT1, AGX1, GFA1, URA2
cal04146	56.56852	Peroxisome	FAA21, CaJ7_0483, AGX1
cal03030	54.2462	DNA replication	PRI1, DPB3
cal00071	54.03803	Fatty acid degradation	FAA21, CaJ7_0483, cal:CaO19.13487, SAD1
cal00061	43.38386	Fatty acid biosynthesis	FAA21, CaJ7_0483, ACC1, FAS1
cal00350	42.3465	Tyrosine metabolism	AAT1, SAD1, AMO2
cal00360	39.59607	Phenylalanine metabolism	AAT1, AMO2
cal00400	36.1654	Phenylalanine, tyrosine and tryptophan biosynthesis	AAT1
cal00270	28.13576	Cysteine and methionine metabolism	AAT1, MDH2
cal00520	24.9472	Amino sugar and nucleotide sugar metabolism	GLK3, GFA1, cal:CaO19.14031
cal00240	23.8103	Pyrimidine metabolism	YND1, PRI1, DPB3, URA2
cal00564	21.70483	Glycerophospholipid metabolism	PSD1, CHO1, OPI3, CRD1, cal:CaO19.12881
cal00030	20.34725	Pentose phosphate pathway	RKI1, PFK2, cal:CaO19.14031
cal00460	12.2906	Cyanoamino acid metabolism	ASP1
cal03420	11.9899	Nucleotide excision repair	DPB3
cal03410	11.9899	Base excision repair	DPB3
cal03018	2.67299	RNA degradation	PFK2
cal00051	−2.79647	Fructose and mannose metabolism	GLK3, PFK2
cal00561	−5.29207	Glycerolipid metabolism	cal:CaO19.13487, cal:CaO19.12881, TGL2
cal00020	−8.02964	Citrate cycle (TCA cycle)	MDH2
cal00565	−8.28607	Ether lipid metabolism	cal:CaO19.12881
cal00100	−8.28607	Steroid biosynthesis	cal:CaO19.12881
cal00590	−8.28607	Arachidonic acid metabolism	cal:CaO19.12881
cal00592	−8.28607	alpha-Linolenic acid metabolism	cal:CaO19.12881
cal00260	−9.61059	Glycine, serine and threonine metabolism	CHO1, AGX1, AMO2
cal00640	−15.0766	Propanoate metabolism	ACS1, ACC1
cal00040	−15.1134	Pentose and glucuronate interconversions	cal:CaO19.13487
cal00280	−15.1134	Valine, leucine and isoleucine degradation	cal:CaO19.13487
cal00053	−15.1134	Ascorbate and aldarate metabolism	cal:CaO19.13487
cal00340	−15.1134	Histidine metabolism	cal:CaO19.13487
cal00310	−15.1134	Lysine degradation	cal:CaO19.13487
cal00380	−15.1134	Tryptophan metabolism	cal:CaO19.13487
cal00680	−16.4076	Methane metabolism	ACS1, AGX1, PFK2
cal00052	−24.6707	Galactose metabolism	GLK3, MAL2, PFK2, cal:CaO19.14031
cal00630	−32.8304	Glyoxylate and dicarboxylate metabolism	MDH2, AGX1, MLS1
cal00010	−36.1765	Glycolysis/Gluconeogenesis	GLK3, ACS1, cal:CaO19.13487, CDC19, SAD1, PFK2, cal:CaO19.14031
cal00620	−59.867	Pyruvate metabolism	ACS1, cal:CaO19.13487, ACC1, CDC19, MDH2, MAE1, MLS1

The cumulative score is the sum of the scores of genes in the pathway that were found in the maximum-scoring sub network.

**Table 3:** Real-time PCR primers used in this study.

Gene	Direction	Primer sequence (5′→3′)
AAT1	Forward	CATTGGCTCCACCAGACAAG
	Reverse	TCTCTATAAGCACCAACCCCC
SAD1	Forward	AGGTCTAGGTGCAACTTCGC
	Reverse	CAGGGTACCCCAGAATGAGC
XOG1	Forward	CCAAGTGTTTTCCGGTGGTG
	Reverse	TCCCAACCCCAGTTACAAGC
ACC1	Forward	TGGAGATTAAGAGTTACTGGTGC
	Reverse	GATAGCACGCAATGGGAACG

## References

[b1] Wisplinghoff H. . Nosocomial bloodstream infections in US hospitals: analysis of 24,179 cases from a prospective nationwide surveillance study. Clin Infect Dis 39, 309–317, doi: 10.1086/421946 (2004). 15306996

[b2] Pfaller M. A. & Diekema D. J. Epidemiology of invasive candidiasis: a persistent public health problem. Clin Microbiol Rev 20, 133–163, doi: 10.1128/CMR.00029-06 (2007). 17223626 PMC1797637

[b3] Ziegler M. J., Pellegrini D. C. & Safdar N. Attributable mortality of central line associated bloodstream infection: systematic review and meta-analysis. Infection 43, 29–36, doi: 10.1007/s15010-014-0689-y (2015). 25331552

[b4] Yousif A., Jamal M. A. & Raad I. Biofilm-based central line-associated bloodstream infections. Adv Exp Med Biol 830, 157–179, doi: 10.1007/978-3-319-11038-7_10 (2015). 25366227

[b5] Eggimann P., Que Y. A., Revelly J. P. & Pagani J. L. Preventing invasive *Candida* infections. Where could we do better? J Hosp Infect 89, 302–308, doi: 10.1016/j.jhin.2014.11.006 (2015). 25592726

[b6] Kollef M., Micek S., Hampton N., Doherty J. A. & Kumar A. Septic shock attributed to *Candida* infection: importance of empiric therapy and source control. Clin Infect Dis 54, 1739–1746, doi: 10.1093/cid/cis305 (2012). 22423135

[b7] Ramage G., Rajendran R., Sherry L. & Williams C. Fungal biofilm resistance. International journal of microbiology 2012, 528521, doi: 10.1155/2012/528521 (2012). 22518145 PMC3299327

[b8] Brown A. J., Brown G. D., Netea M. G. & Gow N. A. Metabolism impacts upon Candida immunogenicity and pathogenicity at multiple levels. Trends Microbiol 22, 614–622, doi: 10.1016/j.tim.2014.07.001 (2014). 25088819 PMC4222764

[b9] Tripathi G. . Gcn4 co-ordinates morphogenetic and metabolic responses to amino acid starvation in *Candida albicans*. EMBO J 21, 5448–5456 (2002). 12374745 10.1093/emboj/cdf507 PMC129063

[b10] Sudbery P. E. Growth of Candida albicans hyphae. Nat Rev Microbiol 9, 737–748, doi: 10.1038/nrmicro2636 (2011). 21844880

[b11] Nobile C. J. & Johnson A. D. Candida albicans Biofilms and Human Disease. Annual review of microbiology 69, 71–92, doi: 10.1146/annurev-micro-091014-104330 (2015). PMC4930275 26488273

[b12] Nobile C. J. . A recently evolved transcriptional network controls biofilm development in Candida albicans. Cell 148, 126–138, doi: 10.1016/j.cell.2011.10.048 (2012). 22265407 PMC3266547

[b13] Garcia-Sanchez S. . *Candida albicans* biofilms: a developmental state associated with specific and stable gene expression patterns. Eukaryotic cell 3, 536–545 (2004). 15075282 10.1128/EC.3.2.536-545.2004 PMC387656

[b14] Lindsay A. K. . Analysis of *Candida albicans* mutants defective in the Cdk8 module of mediator reveal links between metabolism and biofilm formation. PLoS genetics 10, e1004567, doi: 10.1371/journal.pgen.1004567 (2014). 25275466 PMC4183431

[b15] Tumbarello M. . Biofilm production by *Candida* species and inadequate antifungal therapy as predictors of mortality for patients with candidemia. Journal of clinical microbiology 45, 1843–1850, doi: 10.1128/JCM.00131-07 (2007). 17460052 PMC1933062

[b16] Tumbarello M. . Risk factors and outcomes of candidemia caused by biofilm-forming isolates in a tertiary care hospital. PLoS One 7, e33705, doi: 10.1371/journal.pone.0033705 (2012). 22479431 PMC3316499

[b17] Rajendran R. . Biofilm formation is a risk factor for mortality in patients with *Candida albicans* bloodstream infection - Scotland, 2012-2013. *Clinical microbiology and infection: the official publication of the European* Society of Clinical Microbiology and Infectious Diseases, doi: 10.1016/j.cmi.2015.09.018 (2015). PMC4721535 26432192

[b18] Kanehisa M. & Goto S. KEGG: kyoto encyclopedia of genes and genomes. Nucleic acids research 28, 27–30 (2000). 10592173 10.1093/nar/28.1.27 PMC102409

[b19] May A. . metaModules identifies key functional subnetworks in microbiome-related disease. Bioinformatics 32, 1678–1685, doi: 10.1093/bioinformatics/btv526 (2016). 26342232

[b20] Dittrich M. T., Klau G. W., Rosenwald A., Dandekar T. & Muller T. Identifying functional modules in protein-protein interaction networks: an integrated exact approach. Bioinformatics 24, i223–i231, doi: 10.1093/bioinformatics/btn161 (2008). 18586718 PMC2718639

[b21] Kanehisa M. & Goto S. KEGG: Kyoto Encyclopedia of Genes and Genomes. Nucleic acids research 28, 27–30, doi: 10.1093/Nar/28.1.27 (2000). 10592173 PMC102409

[b22] Harris M. A. . The Gene Ontology (GO) database and informatics resource. Nucleic acids research 32, D258–D261, doi: 10.1093/nar/gkh036 (2004). 14681407 PMC308770

[b23] Staab J. F., Datta K. & Rhee P. Niche-specific requirement for hyphal wall protein 1 in virulence of Candida albicans. PLoS One 8, e80842, doi: 10.1371/journal.pone.0080842 (2013). 24260489 PMC3832661

[b24] Nobile C. J., Nett J. E., Andes D. R. & Mitchell A. P. Function of *Candida albicans* adhesin Hwp1 in biofilm formation. Eukaryotic cell 5, 1604–1610, doi: 10.1128/EC.00194-06 (2006). 17030992 PMC1595337

[b25] Granger B. L. Insight into the antiadhesive effect of yeast wall protein 1 of *Candida albicans*. Eukaryotic cell 11, 795–805, doi: 10.1128/EC.00026-12 (2012). 22505336 PMC3370456

[b26] Moyes D. L. . Candidalysin is a fungal peptide toxin critical for mucosal infection. Nature 532, 64–68, doi: 10.1038/nature17625 (2016). 27027296 PMC4851236

[b27] Ramage G., Coco B., Sherry L., Bagg J. & Lappin D. F. *In vitro Candida albicans* biofilm induced proteinase activity and SAP8 expression correlates with *in vivo* denture stomatitis severity. Mycopathologia 174, 11–19, doi: 10.1007/s11046-012-9522-2 (2012). 22302440

[b28] Santana I. L. . Dietary carbohydrates modulate *Candida albicans* biofilm development on the denture surface. PLoS One 8, e64645, doi: 10.1371/journal.pone.0064645 (2013). 23737992 PMC3667795

[b29] Rajendran R. . Extracellular DNA release confers heterogeneity in *Candida albicans* biofilm formation. BMC microbiology 14, 303, doi: 10.1186/s12866-014-0303-6 (2014). 25476750 PMC4262977

[b30] Yeater K. M. . Temporal analysis of *Candida albicans* gene expression during biofilm development. Microbiology 153, 2373–2385, doi: 10.1099/mic.0.2007/006163-0 (2007). 17660402

[b31] Schweizer E. & Hofmann J. Microbial type I fatty acid synthases (FAS): major players in a network of cellular FAS systems. Microbiol Mol Biol Rev 68, 501–517, table of contents, doi: 10.1128/MMBR.68.3.501-517.2004 (2004). 15353567 PMC515254

[b32] Hernandez R., Nombela C., Diez-Orejas R. & Gil C. Two-dimensional reference map of *Candida albicans* hyphal forms. Proteomics 4, 374–382, doi: 10.1002/pmic.200300608 (2004). 14760707

[b33] Xu D. . Genome-wide fitness test and mechanism-of-action studies of inhibitory compounds in *Candida albicans*. PLoS Pathog 3, e92, doi: 10.1371/journal.ppat.0030092 (2007). 17604452 PMC1904411

[b34] Taff H. T. . A *Candida* biofilm-induced pathway for matrix glucan delivery: implications for drug resistance. PLoS Pathog 8, e1002848, doi: 10.1371/journal.ppat.1002848 (2012). 22876186 PMC3410897

[b35] Zara G. . FLO11 expression and lipid biosynthesis are required for air-liquid biofilm formation in a *Saccharomyces cerevisiae* flor strain. FEMS Yeast Res 12, 864–866, doi: 10.1111/j.1567-1364.2012.00831.x (2012). 22805178

[b36] Sherry L. . Biofilms formed by *Candida albicans* bloodstream isolates display phenotypic and transcriptional heterogeneity that are associated with resistance and pathogenicity. BMC microbiology 14, 182, doi: 10.1186/1471-2180-14-182 (2014). 24996549 PMC4105547

[b37] Ramage G., Vande Walle K., Wickes B. L. & Lopez-Ribot J. L. Standardized method for *in vitro* antifungal susceptibility testing of *Candida albicans* biofilms. Antimicrob Agents Chemother 45, 2475–2479 (2001). 11502517 10.1128/AAC.45.9.2475-2479.2001 PMC90680

[b38] Ramage G., Bachmann S., Patterson T. F., Wickes B. L. & Lopez-Ribot J. L. Investigation of multidrug efflux pumps in relation to fluconazole resistance in *Candida albicans* biofilms. J Antimicrob Chemother 49, 973–980 (2002). 12039889 10.1093/jac/dkf049

[b39] Martin M. Cutadapt removes adapter sequences from high-throughput sequencing reads. 2011 17, doi: 10.14806/ej.17.1.200pp.10-12 (2011).

[b40] Kopylova E., Noe L. & Touzet H. SortMeRNA: fast and accurate filtering of ribosomal RNAs in metatranscriptomic data. Bioinformatics 28, 3211–3217, doi: 10.1093/bioinformatics/bts611 (2012). 23071270

[b41] Edgar R. C. Search and clustering orders of magnitude faster than BLAST. Bioinformatics 26, 2460–2461, doi: 10.1093/bioinformatics/btq461 (2010). 20709691

[b42] Dobin A. . STAR: ultrafast universal RNA-seq aligner. Bioinformatics 29, 15–21, doi: 10.1093/bioinformatics/bts635 (2013). 23104886 PMC3530905

[b43] Anders S., Pyl P. T. & Huber W. HTSeq–a Python framework to work with high-throughput sequencing data. Bioinformatics 31, 166–169, doi: 10.1093/bioinformatics/btu638 (2015). 25260700 PMC4287950

[b44] Love M. I., Huber W. & Anders S. Moderated estimation of fold change and dispersion for RNA-seq data with DESeq2. Genome biology 15, 550, doi: 10.1186/s13059-014-0550-8 (2014). 25516281 PMC4302049

[b45] Inglis D. O. . The *Candida* genome database incorporates multiple *Candida* species: multispecies search and analysis tools with curated gene and protein information for *Candida albicans* and *Candida glabrata*. Nucleic acids research 40, D667–D674, doi: 10.1093/nar/gkr945 (2012). 22064862 PMC3245171

[b46] Dittrich M. T., Klau G. W., Rosenwald A., Dandekar T. & Muller T. Identifying functional modules in protein-protein interaction networks: an integrated exact approach. Bioinformatics 24, I223–I231, doi: 10.1093/bioinformatics/btn161 (2008). 18586718 PMC2718639

[b47] May A. . metaModules identifies key functional subnetworks in microbiome-related disease. Bioinformatics, doi: 10.1093/bioinformatics/btv526 (2015). 26342232

[b48] Beisser D., Klau G. W., Dandekar T., Muller T. & Dittrich M. T. BioNet: an R-Package for the functional analysis of biological networks. Bioinformatics 26, 1129–1130, doi: 10.1093/bioinformatics/btq089 (2010). 20189939

[b49] Pounds S. & Morris S. W. Estimating the occurrence of false positives and false negatives in microarray studies by approximating and partitioning the empirical distribution of p-values. Bioinformatics 19, 1236–1242, doi: 10.1093/bioinformatics/btg148 (2003). 12835267

[b50] Shannon P. . Cytoscape: A software environment for integrated models of biomolecular interaction networks. Genome Res 13, 2498–2504, doi: 10.1101/Gr.1239303 (2003). 14597658 PMC403769

[b51] Dinkla K. . eXamine: exploring annotated modules in networks. BMC bioinformatics 15, 201, doi: 10.1186/1471-2105-15-201 (2014). 25002203 PMC4084410

[b52] Erlandsen S. L., Kristich C. J., Dunny G. M. & Wells C. L. High-resolution visualization of the microbial glycocalyx with low-voltage scanning electron microscopy: dependence on cationic dyes. J Histochem Cytochem 52, 1427–1435, doi: 10.1369/jhc.4A6428.2004 (2004). 15505337 PMC3957825

