# Supplementary material for: Integrating Candida albicans metabolism with biofilm heterogeneity by transcriptome mapping
Source: Sci Rep. 2016 Oct 21;6:35436. doi: 10.1038/srep35436 (PMC5073228; doi:10.1038/srep35436)
Supplement: Supplementary Information [file srep35436-s1.doc]

**Integrating *Candida albicans* metabolism with biofilm heterogeneity by transcriptome mapping**

Ranjith Rajendran^1^**^§^**, Ali May^2,3^**^§^**, Leighann Sherry^1^, Ryan Kean^1,4^, Craig Williams^4^, Brian Jones^5^, Karl Burgess^6^, Jaap Heringa^3^, Sanne Abeln^3^, Bernd W. Brandt^2^, Carol Munro^7^ and Gordon Ramage^1^*

^1^School of Medicine, College of Medical, Veterinary and Life Sciences (MVLS), University of Glasgow, UK, ^2^Department of Preventive Dentistry, Academic Centre for Dentistry Amsterdam (ACTA), University of Amsterdam and VU University Amsterdam, The Netherlands, ^3^Centre for Integrative Bioinformatics VU (IBIVU), VU University Amsterdam, The Netherlands, ^4^Institute of Healthcare Associated Infection, School of Health, Nursing and Midwifery, University of the West of Scotland, UK, ^5^Microbiology Department, Glasgow Royal Infirmary, Glasgow, UK, ^6^Polyomics Facility, MVLS, University of Glasgow, UK, ^7^Aberdeen Fungal Group, MRC Centre for Medical Mycology, University of Aberdeen, UK.

**^§^**Both authors contributed equally to the manuscript.

*Corresponding Author: Gordon Ramage, Oral Sciences Research Group, School of Medicine, Dentistry and Nursing, College of Medical, Veterinary and Life Sciences, University of Glasgow, 378 Sauchiehall Street, Glasgow, G2 3JZ, UK. Phone: +44(0)141 211 9752. Fax: +44(0) 141 331 2798. e-mail: [gordon.ramage@glasgow.ac.uk](mailto:gordon.ramage@glasgow.ac.uk)


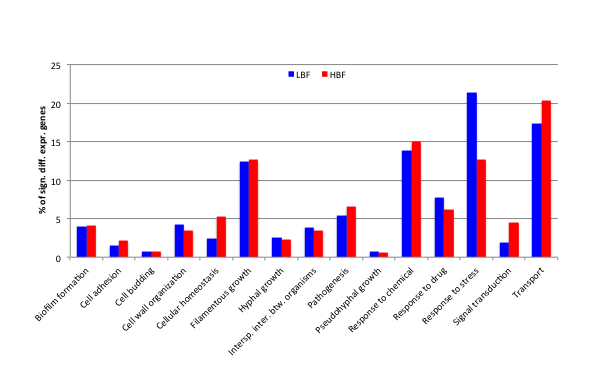


**Supplementary Figure 1:** Bar  chart showing the percentage of genes that were upregulated by a minimum 2-  fold change (P < 0.05) for each functional category in the LBF (blue) and HBF (red).

Supplementary Figure 2. A beta-uniform mixture model (not shown) was fit to the *P* value distribution of gene fold changes (the primary axis), based on which a numerical score was calculated for each gene in the global *C. albicans* network (the secondary axis). Here, genes with smaller *P* values than the *P* value threshold (10^-10^) determined by the false-discovery rate (10^-9^) attain positive scores, while those with larger *P* values are assigned negative scores.

**Supplementary figure 3:** Impact of AOA (Aminoxy acetate [AAT inhibitor]) on low biofilm formers. LBF isolates were incubated in the presence of serially diluted AOA (0 - 400 mg/L) for 24h at 37^o^C. Then biofilm biomass assessed by crystal violet assay. No significant change in biofilm biomass observed at all tested concentrations compared to untreated control.

Supplementary Table. The subnetwork identified in the global *C. albicans* network consisted of 39 genes.

| Gene ID | Network (node) score | *P*-value | Log_2_FC (LBF/HBF) |
| --- | --- | --- | --- |
| AAT1 | 36.2 | 1.0E-27 | -2.8 |
| ACC1 | -5.8 | 8.2E-08 | -1.9 |
| ACS1 | -9.2 | 3.4E-06 | -2.9 |
| AFP98 | 47.0 | 7.4E-33 | -2.8 |
| AFP99 | 14.2 | 2.5E-17 | -2.6 |
| AGX1 | -9.8 | 6.5E-06 | -1.5 |
| AMD1 | 106.9 | 3.0E-61 | 2.3 |
| AMO2 | 3.4 | 3.4E-12 | -1.2 |
| ASP1 | 12.3 | 2.1E-16 | -1.8 |
| CaJ7_0483 | 52.7 | 1.5E-35 | -1.9 |
| cal:CaO19.12881 | -8.3 | 1.2E-06 | -1.0 |
| cal:CaO19.13487 | -15.1 | 2.1E-03 | -0.4 |
| cal:CaO19.14031 | -4.0 | 1.1E-08 | 0.9 |
| CBP1 | 116.7 | 7.3E-66 | -2.5 |
| CDC19 | -7.8 | 7.0E-07 | 1.9 |
| CHO1 | -3.2 | 4.7E-09 | 1.2 |
| CRD1 | 42.3 | 1.3E-30 | 2.6 |
| DPB3 | 12.0 | 3.0E-16 | 1.8 |
| FAA21 | 13.7 | 4.5E-17 | -2.3 |
| FAS1 | -17.2 | 2.0E-02 | -1.5 |
| GDB1 | -4.5 | 1.9E-08 | 1.3 |
| GFA1 | 34.4 | 7.2E-27 | 1.1 |
| GLK3 | -5.5 | 5.5E-08 | 2.1 |
| GSY1 | -11.5 | 3.9E-05 | 0.9 |
| MAE1 | 1.1 | 4.2E-11 | 2.5 |
| MAL2 | -17.9 | 4.3E-02 | 0.7 |
| MDH2 | -8.0 | 9.1E-07 | -2.6 |
| MLS1 | -15.0 | 1.8E-03 | -1.7 |
| OPI3 | 5.6 | 3.2E-13 | -1.7 |
| PFK2 | 2.7 | 7.7E-12 | 1.2 |
| PRI1 | 42.3 | 1.3E-30 | 2.1 |
| PSD1 | -14.7 | 1.3E-03 | 0.6 |
| RKI1 | 21.6 | 7.8E-21 | -2.7 |
| SAD1 | 2.8 | 7.1E-12 | 4.4 |
| TGL2 | 18.1 | 3.7E-19 | 1.7 |
| TPS2 | 28.1 | 6.7E-24 | -1.2 |
| URA2 | -13.2 | 2.5E-04 | 1.5 |
| XOG1 | 126.0 | 2.7E-70 | 4.5 |
| YND1 | -17.3 | 2.1E-02 | -0.3 |
